# Rethinking the distinction between job burnout and depression: the mediating role of stress among healthcare professionals in Ecuador

**DOI:** 10.3389/fpsyg.2025.1516762

**Published:** 2025-06-05

**Authors:** María Aranzazu Cisneros-Vidal, Mateo Peñaherrera-Aguirre, Silvia L. Vaca-Gallegos, Belén Paladines, Pablo Ruisoto

**Affiliations:** ^1^Department of Psychology, Universidad Técnica Particular de Loja, Loja, Ecuador; ^2^School of Animal and Comparative Biomedical Sciences, The University of Arizona, Tucson, AZ, United States; ^3^Department of Health Sciences, Public University of Navarre, Pamplona, Spain; ^4^IdiSNA, Navarra Institute for Health Research, Pamplona, Spain; ^5^I-Communitas, Institute for Advanced Social Research, Pamplona, Spain

**Keywords:** psychological stress, job burnout, depression, healthcare professionals, structural equation modeling, confirmatory factor analysis, measurement invariance

## Abstract

**Background:**

The distinction between job burnout and depression remains debated, particularly among healthcare professionals exposed to chronic work-related stress. This study examined the mediating role of psychological stress in the relationship between job burnout and depression in an Ecuadorian context.

**Methods:**

A cross-sectional sample of 437 physicians and nurses was recruited with the support of Ecuadorian Ministry of Public Health. Participants completed the PHQ-9, the Perceived Stress Scale (PSS-10), and the Spanish version of the Maslach Burnout Inventory–Human Services Survey (MBI-HSS). Confirmatory factor analysis was used to assess the validity of all instruments. A structural equation model tested the mediating effect of psychological stress, and multi-group analyses evaluated measurement invariance by gender.

**Results:**

Confirmatory factor analysis supported the latent structures of the PSS-10 and PHQ-9, while only the personal accomplishment subscale of the MBI-HSS demonstrated satisfactory psychometric properties in this sample. The structural equation model showed that psychological stress partially mediated the association between personal accomplishment and depressive symptoms. Measurement invariance across gender was supported, although males and females exhibited different symptom patterns: Men reported more cognitive-affective symptoms, while women endorsed more somatic and concentration-motor features.

**Conclusion:**

These findings support the interpretation of job burnout as a potential work-related manifestation of depression, with psychological stress functioning as a transdiagnostic mechanism. From a public health perspective, interventions should target not only individual-level symptoms but also systemic occupational stressors that shape mental health outcomes among healthcare professionals.

## Background

The distinction between job burnout and depression has long been a subject of debate and importance in the field of psychology. Burnout was first described by Freudenberger in 1974, who characterized it as someone who “looks, acts, and seems depressed” (pp. 161) (Freudenberger, 1974). Emotional exhaustion, depersonalization, and a lack of professional efficacy later became the core dimensions of job burnout, as formalized by Maslach et al. (2001). However, these dimensions have been shown to overlap substantially with the symptoms of depression, leading to questions about their conceptual independence (Bianchi et al., 2022; Bianchi et al., 2014; Bianchi et al., 2015).

Despite extensive research, no consensus has emerged regarding whether job burnout constitutes a distinct syndrome or a specific manifestation of depression resulting from work-related stress (Bianchi et al., 2014; Akil, 2005; Danhof-Pont et al., 2011). The International Classification of Diseases (ICD-11) defines burnout as an occupational phenomenon rather than a mental disorder, while the Diagnostic and Statistical Manual of Mental Disorders (DSM-5) does not recognize it as a clinical diagnosis.

This debate is particularly relevant in healthcare settings, where job burnout is widespread and often co-occurs with depressive symptoms (Gold, 2015; Hammen, 2005). In some cases, job burnout may mask underlying depressive disorders, leading to delays in diagnosis and treatment. This is especially concerning given that job burnout has been associated with increased risks of insomnia (Armon et al., 2008), cardiovascular disease (Toker et al., 2012), hospitalization (Toppinen-Tanner et al., 2009), and even premature mortality (Ahola et al., 2012; Ahola et al., 2010).

Adding to the complexity is the continued reliance on the Maslach Burnout Inventory–Human Services Survey (MBI-HSS) (Schaufeli et al., 2009), which has been criticized for its psychometric limitations and lack of cross-cultural consistency (Cox et al., 2005; Shirom and Melamed, 2006). Of the three MBI-HSS subscales, personal accomplishment has shown the most consistent psychometric performance, while emotional exhaustion and depersonalization have demonstrated reduced reliability in some contexts (Schaufeli et al., 2009; Cox et al., 2005; Danhof-Pont et al., 2011).

Psychological stress is increasingly viewed as a central explanatory factor in both job burnout and depression (Hammen, 2005; Kakiashvili et al., 2013). Chronic exposure to stressors can impair neuroendocrine regulation and contribute to emotional dysregulation, cognitive impairment, and learned helplessness—core components in both syndromes (Gold, 2015; Rössler et al., 2015).

Understanding the potential mediating role of psychological stress may help clarify the relationship between job burnout and depression. This study investigates that relationship in a sample of Ecuadorian healthcare professionals—a group facing high levels of structural vulnerability and chronic occupational stress. Specifically, we use structural equation modeling to test whether psychological stress mediates the association between personal accomplishment and depressive symptoms, while also examining measurement invariance by gender.

## Methods

### Participants

A non-probabilistic, non-clinical sample of 663 healthcare professionals (physicians and nurses) from the Southern region of Ecuador was invited to participate in an online survey, disseminated with the support of the Ministry of Public Health. Data collection was carried out over 4 months (November–February 2022). Participants provided written consent, and no financial incentives were offered. After excluding incomplete responses, the final sample was composed of 437 healthcare professionals (318 women and 119 men), with a mean age of 34.16 years (SD = 9.48, range = 18–72). Of these, 57.2% were nurses and 42.8% were physicians. Additional variables recorded included employment status, geographic region, average daily working hours, and marital status.

### Measures

The sociodemographic questionnaire included age, sex, marital status, geographic region in Ecuador, employment status, and average number of hours/day.

Job burnout was assessed using the Spanish version of the Maslach Burnout Inventory–Human Services Survey (MBI-HSS) (Maslach and Jackson, 1996). While no Ecuador-specific adaptation exists, it is the most widely used instrument for assessing job burnout and includes three subscales: (1) emotional exhaustion, (2) depersonalization, and (3) personal accomplishment. The Spanish version of the MBI-HSS was used in this study. Each item is rated on a 7-point Likert scale ranging from 0 (“Never”) to 6 (“Every day”), referring to the past year. Higher scores in emotional exhaustion and depersonalization indicate higher burnout, while lower scores in personal accomplishment indicate greater burnout. An overall scale in burnout is calculated from all three scales. An example item is “I feel emotionally drained from my work.” Psychometric validation was conducted through confirmatory factor analysis (CFA), and internal consistency was assessed using Cronbach’s *α* and McDonald’s *ω*.

Perceived stress was assessed using the 10-item Perceived Stress Scale (PSS-10) (Cohen et al., 1983). The Spanish version validated for Ecuadorian healthcare professionals was used (Ruisoto et al., 2020). The PSS-10 includes positively and negatively worded items. Each item is rated on a 5-point Likert scale, ranging from 0 (“never”) to 4 (“very often”), referring to the last month. Higher scores indicate higher levels of perceived stress.

Depressive symptoms were measured using the Patient Health Questionnaire-9 (PHQ-9) (Kroenke et al., 2001), based on the diagnostic criteria of the DSM. The PHQ-9 includes nine items rated on a 4-point Likert scale, ranging from 0 (“not at all”) to 3 (“nearly every day”), referring to the past 2 weeks. Higher scores indicate more severe depressive symptomatology. The Spanish version validated in Ecuador was used (López-Guerra et al., 2022).

### Design and procedure

This was a cross-sectional study conducted with ethical approval from the Ethics Committee for Research in Human Beings at the Universidad of Cuenca (Approval code: 2022-020EO-IE). This study was supported by the Ecuadorian Ministry of Public Health, which facilitated participant recruitment. All participants provided informed consent in accordance with the guidelines of the Declaration of Helsinki (World Medical Association, 2013). Data were collected online between November 2022 and February 2023.

### Statistical analyses

Statistical analyses were conducted in two stages. First, confirmatory factor analysis (CFA) was used to examine the psychometric structure of PSS-10, PHQ-9, and MBI-HSS (Benach et al., 2014). These analyses assessed model fit using the comparative fit index (CFI), Tucker–Lewis Index (TLI), root mean square error of approximation (RMSEA), and standardized root mean square residual (SRMR). Internal consistency was evaluated using Cronbach’s *α* and McDonald’s *ω*, with results reported for each subscale.

In the second stage, a structural equation model (SEM) was specified to examine whether psychological stress mediated the relationship between personal accomplishment (MBI-HSS) and depressive symptoms (PHQ-9). Factor scores were computed using unit-weighted methods for each scale’s relevant subdimensions. Multi-group SEM was also performed to test measurement invariance by gender, including configural, metric, scalar, residual, structural, and strict invariance levels.

All analyses were conducted using R version 4.0.1, using the *psych* (Revelle, 2015), *lavaan* (Rosseel, 2012), and *semTools* (Jorgensen et al., 2022) packages.

## Results

### Sample description

The final sample consisted of 437 healthcare professionals (318 women and 119 men), with a mean age of 34.16 years (SD = 9.48; range = 18–72). Of these, 52.9% were nurses and 47.1% were physicians. Additional sociodemographic variables were recorded, including marital status (52.4% single, 41.4% married, 5.3% divorced/separated, and 0.9% widowed), employment status, region, and average daily work hours.

### Measurement models

We conducted confirmatory factor analysis (CFA) to assess the psychometric structure and reliability of the PHQ-9, PSS-10, and MBI-HSS.

#### PHQ-9

The CFA supported a higher-order model of depression with three first-order latent factors: somatic, cognitive-affective, and concentration-motor dimensions. Fit indices indicated a good model fit: *χ*^2^_(24)_ = 94.97, *p* < 0.001; CFI = 0.971; TLI = 0.957; RMSEA = 0.083; SRMR = 0.031. Internal consistency was adequate across all subscales (*α* and *ω* values between 0.79 and 0.88). Table 1 presents the standardized factor loadings for the PHQ-9 items.

**Table 1:** Measurement model based on a confirmatory factor analysis for PHQ items.

Variables	Standardized estimate	Standard error	*z*-value	*p*-value
Somatic				
PHQ-3	0.749	0.058	3.774	0.0000
PHQ-4	0.821	0.061	3.654	0.0000
PHQ-5	0.832	0.064	3.777	0.0000
Cognitive-affective				
PHQ-1	0.787	0.042	5.321	0.0000
PHQ-2	0.852	0.049	5.203	0.0000
PHQ-6	0.828	0.044	5.343	0.0000
PHQ-9	0.706	0.036	4.903	0.0000
Concentration-motor				
PHQ-7	0.862	0.057	4.617	0.0000
PHQ-8	0.760	0.045	4.646	0.0000
Depression				
Somatic	0.951	0.882	3.479	0.0010
Cognitive-affective	0.937	0.552	4.853	0.0000
Concentration-motor	0.923	0.552	4.343	0.0000

#### PSS-10

CFA results for the PSS-10 supported a two-factor structure distinguishing positive and negative stress. Model fit was acceptable: *χ*^2^_(34)_ = 145.43, *p* < 0.001; CFI = 0.937; TLI = 0.917; RMSEA = 0.087; SRMR = 0.063. Internal consistency was strong for the negative factor (*α* = 0.88; *ω* = 0.88) and adequate for the positive factor (*α* = 0.76; *ω* = 0.75). Table 2 provides standardized loadings and model estimates for the PSS-10.

**Table 2:** Measurement model based on a confirmatory factor analysis for PSS items.

Variables	Standardized estimate	Standard error	*z-*value	*p*-value
Negative				
PSS1	0.678	0.045	14.153	0.0000
PSS2	0.771	0.041	18.066	0.0000
PSS3	0.733	0.036	18.684	0.0000
PSS6	0.717	0.042	14.51	0.0000
PSS9	0.690	0.040	15.328	0.0000
PSS10	0.846	0.038	21.734	0.0000
Positive				
PSS4	0.536	0.053	9.225	0.0000
PSS5	0.698	0.050	12.655	0.0000
PSS7	0.713	0.045	12.84	0.0000
PSS8	0.687	0.042	13.677	0.0000

Covariance	Standardized estimate	Standard error	*z*-value	*p*-value
Negative				
Positive	−0.605	0.064	−9.436	0.0000

#### MBI-HSS

A bifactor model was tested for the Maslach Burnout Inventory-Human Services Survey (MBI-HSS). Although the global fit was acceptable [*χ*^2^_(184)_ = 510.45, *p* < 0.001; CFI = 0.932; TLI = 0.915; RMSEA = 0.064; SRMR = 0.040], only the Personal Accomplishment subscale demonstrated sufficient internal consistency (*α* = 0.85; *ω* = 0.79). The emotional exhaustion and depersonalization subscales had low *ω* values (0.07 and 0.53, respectively) and were excluded from further analysis. Table 3 shows the factor loadings and psychometric properties for the MBI-HSS subscales.

**Table 3:** Measurement model based on confirmatory factor analysis for MBI-HSS items.

Variables	Standardized estimate	Standard error	*z*-value	*p*-value
Burnout				
MBI-HSS1	0.875	0.055	29.479	0.0000
MBI-HSS2	0.891	0.057	30.019	0.0000
MBI-HSS3	0.883	0.058	30.038	0.0000
MBI-HSS6	0.555	0.178	4.926	0.0000
MBI-HSS8	0.839	0.079	20.024	0.0000
MBI-HSS13	0.705	0.138	8.839	0.0000
MBI-HSS14	0.660	0.137	8.804	0.0000
MBI-HSS16	0.535	0.182	4.660	0.0000
MBI-HSS20	0.473	0.138	6.716	0.0000
MBI-HSS5	0.313	0.101	3.056	0.0020
MBI-HSS10	0.275	0.174	2.913	0.0040
MBI-HSS11	0.490	0.17	5.213	0.0000
MBI-HSS15	0.179	0.143	1.892	0.0580
MBI-HSS22	0.388	0.14	3.930	0.0000
MBI-HSS4	0.011	0.13	0.133	0.8940
MBI-HSS7	0.011	0.116	0.177	0.8600
MBI-HSS9	−0.055	0.134	−0.709	0.4780
MBI-HSS12	−0.537	0.104	−8.937	0.0000
MBI-HSS17	−0.105	0.112	−1.598	0.1100
MBI-HSS18	−0.347	0.105	−5.172	0.0000
MBI-HSS19	−0.231	0.092	−3.646	0.0000
MBI-HSS21	−0.194	0.116	−3.215	0.0010
Emotional exhaustion				
MBI-HSS1	0.070	0.342	0.383	0.7010
MBI-HSS2	0.117	0.372	0.601	0.5480
MBI-HSS3	0.012	0.312	0.074	0.9410
MBI-HSS6	−0.506	0.195	−4.096	0.0000
MBI-HSS8	−0.150	0.257	−1.102	0.2700
MBI-HSS13	−0.343	0.21	−2.838	0.0050
MBI-HSS14	−0.325	0.228	−2.605	0.0090
MBI-HSS16	−0.519	0.193	−4.275	0.0000
MBI-HSS20	−0.253	0.198	−2.497	0.0130
Depersonalization				
MBI-HSS5	0.583	0.097	5.929	0.0000
MBI-HSS10	0.567	0.118	8.841	0.0000
MBI-HSS11	0.507	0.173	5.280	0.0000
MBI-HSS15	0.480	0.106	6.827	0.0000
MBI-HSS22	0.467	0.149	4.440	0.0000
Personal accomplishment				
MBI-HSS4	0.578	0.095	9.773	0.0000
MBI-HSS7	0.586	0.093	12.175	0.0000
MBI-HSS9	0.671	0.093	12.385	0.0000
MBI-HSS12	0.416	0.126	5.757	0.0000
MBI-HSS17	0.667	0.088	13.011	0.0000
MBI-HSS18	0.727	0.099	11.413	0.0000
MBI-HSS19	0.717	0.099	10.491	0.0000
MBI-HSS21	0.599	0.088	13.071	0.0000

### Structural equation model

**Figure 1:**
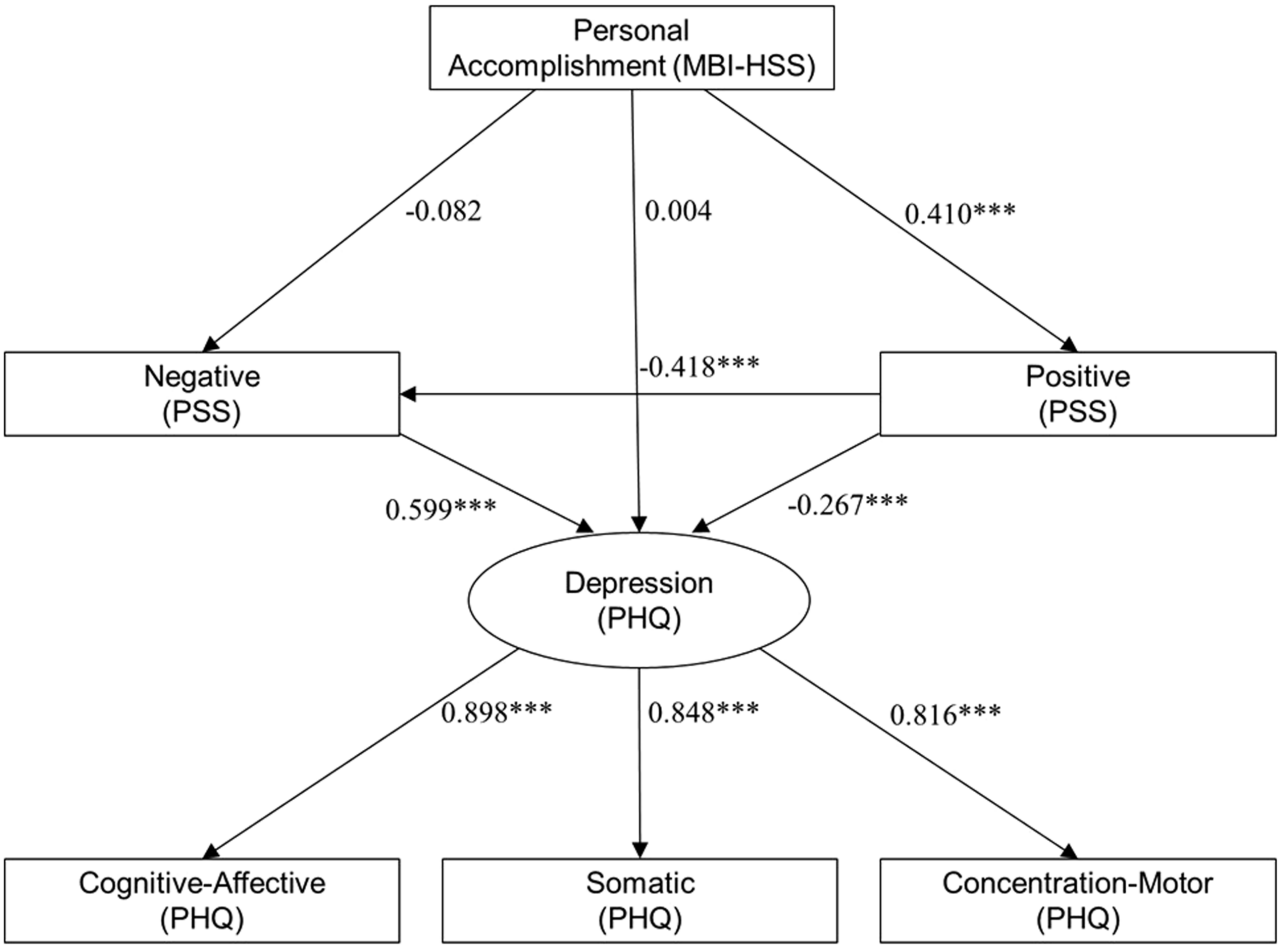
Structural equation model examining the influence of the MBI-HSS scale of personal accomplishment on PSS-based stress scores and PHQ-based depression scores. ***p < 0.0001.

We tested a structural equation model (SEM) to examine whether psychological stress mediated the relationship between personal accomplishment and depression. The model demonstrated excellent fit: *χ*^2^_(8)_ = 14.50, *p* = 0.024; CFI = 0.993; TLI = 0.983; RMSEA = 0.057; SRMR = 0.016. Personal accomplishment positively predicted positive stress. Positive stress negatively predicted depression. Negative stress positively predicted depression. Figure 1 depicts the structural equation model with standardized path coefficients.

### Multi-group analysis by gender

**Figure 2:**
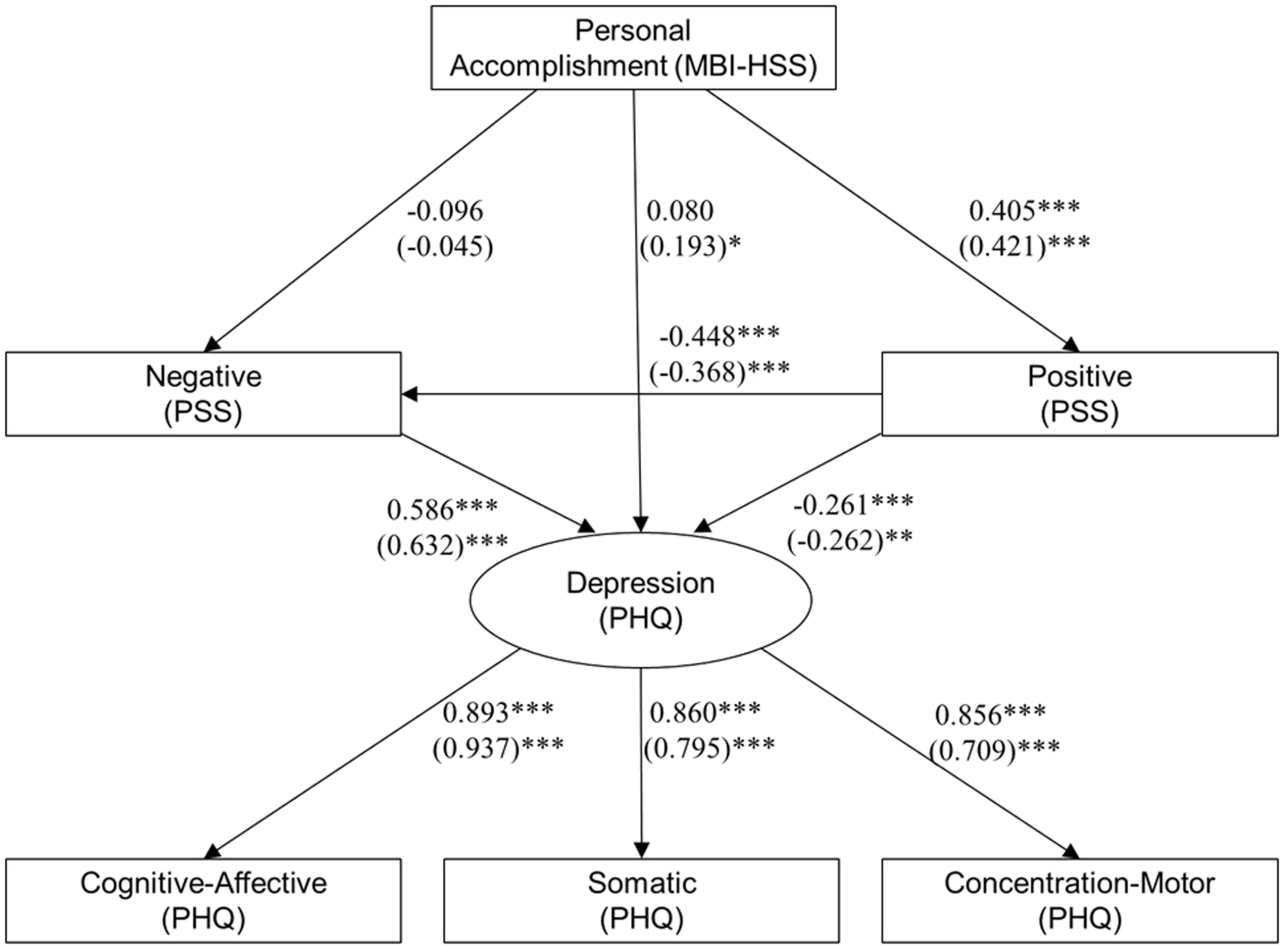
Structural equations model examining the influence of the MBI-HSS scale of personal accomplishment on PSS-based stress scores and PHQ-based depression scores, freeing the parameter estimates between males and females. Values inside the parentheses correspond to the standardized estimates for males. ***p < 0.0001, **p < 0.01, and *p < 0.05.

A multi-group structural equation model was conducted to assess measurement invariance across gender. Model comparisons supported configural, metric, and scalar invariance, indicating that the basic structure, factor loadings, and item intercepts were equivalent for male and female participants. Although residual invariance was only partially met, no significant differences were observed in latent means or covariances, supporting the generalizability of the model across gender groups (Figure 2 and Table 4).

**Table 4:** Model comparison based on participants’ sex, examining the statistical fit across various invariance configurations.

Model	Df	AIC	BIC	X^2^	X^2^ diff	RMSEA	Df diff	*p*-value
Configural invariance	12	4107.300	4261.700	24.975				
Metric invariance	14	4112.600	4258.900	34.294	9.319	0.130	2	0.0095
Scalar invariance	18	4113.100	4243.100	42.750	8.456	0.072	4	0.0762
Residual invariance	23	4118.800	4228.500	58.486	15.737	0.100	5	0.0076
Structural invariance	24	4117.800	4223.500	59.526	1.039	0.013	1	0.3080
Strict invariance	25	4117.600	4219.200	61.280	1.754	0.059	1	0.1854

The results of the multi-group SEM, in which loadings were freed for male and female groups, showed an adequate statistical fit (*χ*^2^ = 24.975, *p* = 0.015; CFI = 0.990; TLI = 0.975; RMSEA = 0.071; SRMR = 0.019). Personal accomplishment did not significantly predict the PSS negative scale in either group. In contrast, a significant positive association was observed between personal accomplishment and the PSS positive scale in both groups. Among males, personal accomplishment also positively influenced overall depression scores, although the effect was small in magnitude. In both groups, negative and positive stress were positively and negatively associated with depression, respectively, with slightly stronger effects observed among males. The depression latent factor loaded positively onto cognitive-affective, somatic, and concentration-motor dimensions, with males showing a stronger loading on cognitive-affective symptoms, while females showed stronger loadings for somatic and concentration-motor dimensions.

## Discussion

The present study examined the relationship between job burnout and depression among healthcare professionals, with particular attention to the mediating role of psychological stress. Our findings underscore the centrality of stress in both phenomena and suggest that the current conceptual distinction between burnout and depression may be fragile, particularly from a parsimony perspective. This aligns with prior evidence showing that both conditions are associated with common adverse outcomes (Armon et al., 2008; Toker et al., 2012; Toppinen-Tanner et al., 2009; Ahola et al., 2012; Ahola et al., 2010), thereby reinforcing the public health relevance of burnout as more than an occupational inconvenience.

Although depression is formally classified as a mental disorder in the DSM-5 and is projected to become one of the leading causes of global disability (World Health Organization, 2023), burnout is defined in the ICD-11 only as an occupational phenomenon, lacking formal diagnostic status. Referring to individuals as “burned out” rather than “depressed” may serve as a stigma-avoidance strategy, particularly in workplace and clinical contexts, which could in turn delay appropriate diagnosis and treatment (Chiu et al., 2015).

Our results have also shown that only the personal accomplishment dimension of the Maslach Burnout Inventory-Human Services Survey (MBI-HSS) met acceptable psychometric standards, consistent with longstanding critiques of the MBI’s factorial and conceptual structure across occupational and cultural contexts (Schaufeli et al., 2009; Cox et al., 2005).

The mediating role of stress provides a theoretically and biologically plausible explanation for the burnout–depression continuum. Chronic stress has been repeatedly linked to depressive symptomatology through neuroendocrine dysregulation, including cortisol variability and HPA axis disturbance (Akil, 2005; Gold, 2015; Hammen, 2005). Our model supports this connection, showing that both positive and negative stress dimensions were significantly associated with depression outcomes, and that stress mediated the relationship between personal accomplishment and depressive symptoms.

The model also demonstrated measurement invariance by gender, suggesting that the job burnout–stress–depression pathway is structurally similar for both men and women. Nonetheless, our multi-group analysis indicated some gendered variation in symptom presentation: males were more likely to report cognitive-affective symptoms, whereas females more frequently endorsed somatic and concentration-motor dimensions.

These findings should be interpreted within the broader context of occupational and socioeconomic conditions. The relationship between precarious employment, elevated stress levels, and adverse mental health outcomes is well documented across Europe (Benach et al., 2014; Bolibar et al., 2021). In Ecuador, healthcare professionals are particularly affected by structural vulnerabilities such as temporary contracts, workforce shortages, and inconsistent employment protections. These factors may amplify their exposure to occupational stress, thereby increasing the risk of both job burnout and depression.

From a public health and policy perspective, our findings reinforce the view that job burnout should not be understood merely as an individual psychological issue but rather as a systemic consequence of occupational exposure to stressors. This supports calls to reframe job burnout as a work-related form of depression, which would legitimize organizational and policy-level interventions such as workload regulation, job design, and psychosocial risk prevention (Benach, 2023; Ahola et al., 2009; Ahola et al., 2008).

Our results also resonate with Geoffrey Rose’s seminal distinction between “sick individuals” and “sick populations” (Rose, 1985). While individual symptoms of burnout or depression may be clinically addressed, the broader preventive potential lies in reducing structural exposures—such as job insecurity, overwork, and under-resourced systems—that disproportionately affect entire groups. Population-level strategies addressing the social determinants of mental health are therefore essential to prevent recurrence and reduce the burden across the healthcare workforce.

### Limitations

Finally, this study has several limitations. First, its cross-sectional design precludes causal inferences. Longitudinal studies are needed to assess the temporal dynamics between stress, job burnout, and depression. Second, all data were obtained via self-report instruments, which may be subject to recall and social desirability biases. Third, although validated Spanish-language instruments were used for depression and stress (Ruisoto et al., 2020; López-Guerra et al., 2022), the MBI-HSS has not been formally adapted to Ecuadorian Spanish. Although CFA provided support for the personal accomplishment subscale, caution is warranted when interpreting job burnout as a whole. Finally, future studies should model potentially relevant variables such as employment type, tenure, or institutional context, which may interact with stress and mental health outcomes in meaningful ways.

## Conclusion

This study provides empirical support for the hypothesis that psychological stress mediates the relationship between job burnout and depression among healthcare professionals. Using validated instruments and a structural equation modeling approach, we found that both positive and negative stress dimensions significantly influence depressive symptomatology and partially explain the association between personal accomplishment and depression. These findings contribute to growing evidence that burnout and depression may not be categorically distinct, but instead lie along a continuum of stress-related psychopathology.

Our results also underscore the psychometric limitations of existing burnout measures—particularly the tripartite structure of the Maslach Burnout Inventory—which showed poor reliability for emotional exhaustion and depersonalization in this context. In contrast, the personal accomplishment subscale emerged as a robust and meaningful construct within our Ecuadorian sample, highlighting the need for context-sensitive validation when applying standardized instruments across cultures.

Beyond individual symptoms, the study points to the importance of addressing the structural determinants of occupational mental health, including precarious employment conditions, institutional stressors, and gendered patterns in symptom expression. These findings support a conceptual reframing of burnout as a work-related form of depression, shaped by chronic exposure to uncontrollable psychosocial stressors.

From a public health perspective, these results echo Geoffrey Rose’s call to address not only “sick individuals,” but the “sick populations” that produce them. Reducing the incidence of burnout and depression among healthcare workers requires moving beyond clinical diagnosis and individual coping strategies to implement population-level interventions aimed at minimizing systemic sources of stress and inequality in the workplace. Such structural approaches are essential not only to protect the mental health of healthcare professionals but also to safeguard the resilience of the health systems that rely on them.

## Data Availability

The raw data supporting the conclusions of this article will be made available by the authors, without undue reservation.
